# *Lepidium peruvianum* as a Source of Compounds with Anticancer and Cosmetic Applications

**DOI:** 10.3390/ijms251910816

**Published:** 2024-10-08

**Authors:** Dorota Kasprzak, Katarzyna Gaweł-Bęben, Wirginia Kukula-Koch, Marcelina Strzępek-Gomółka, Anna Wawruszak, Sylwia Woźniak, Marcelina Chrzanowska, Karolina Czech, Julia Borzyszkowska-Bukowska, Kazimierz Głowniak, Dariusz Matosiuk, Rita Cristina Orihuela-Campos, Barbara Jodłowska-Jędrych, Tomasz Laskowski, Henry O. Meissner

**Affiliations:** 1 Department of Cosmetology, Faculty of Health Sciences, Wincenty Pol Academy of Applied Sciences in Lublin, Choiny 2 Street, 20-816 Lublin, Poland; bimmer.dorota@gmail.com; 2 Department of Cosmetology, The University of Information Technology and Management in Rzeszow, Sucharskiego 2, 35-225 Rzeszow, Poland; kagawel@wsiz.edu.pl (K.G.-B.); marcys.strzepek@gmail.com (M.S.-G.); kczech@wsiz.edu.pl (K.C.); kglowniak@wsiz.edu.pl (K.G.); 3 Department of Pharmacognosy with Medicinal Plants Garden, Medical University of Lublin, 1 Chodzki Str., 20-093 Lublin, Poland; 4 Department of Biochemistry and Molecular Biology, Medical University of Lublin, 1 Chodzki Str., 20-093 Lublin, Poland; 5 Chair and Department of Synthesis and Chemical Technology of Pharmaceutical Substances, Medical University of Lublin, 4a Chodzki Str., 20-93 Lublin, Poland; sylwia.wozniak@umlub.pl (S.W.); dariusz.matosiuk@umlub.pl (D.M.); 6 Department of Pharmaceutical Technology and Biochemistry and BioTechMed Centre, Faculty of Chemistry, Gdańsk University of Technology, Gabriela Narutowicza 11/12 St., 80-233 Gdańsk, Poland; chrzanowskampoczta@gmail.com (M.C.); julia.borzyszkowska-bukowska@pg.edu.pl (J.B.-B.); tomasz.laskowski@pg.edu.pl (T.L.); 7 Academic Department of Stomatology for Children and Adolescents, Integrated Faculties of Medicine, Stomatology and Nursing, Cayetano Heredia Peruvian University, Av. Honorio Delgado 430, Lima 15102, Peru; rita.orihuela.c@upch.pe; 8 Department of Histology and Embryology, Medical University of Lublin, 11 Radziwiłłowska Str., 20-093 Lublin, Poland; b.jedrych@gmail.com; 9 Therapeutic Research, TTD International Pty Ltd., 39 Leopard Ave., Elanora, Gold Coast, QLD 4221, Australia; dr.meissner@ttdintnl.com.au

**Keywords:** *Lepidium meyenii*, glucotropaeolin, cancer, cosmetics, antioxidants, tyrosinase inhibitors, glucosinolanes, skin

## Abstract

*Lepidium peruvianum*—an edible herbaceous biennial plant distributed in the Andes—has been used for centuries as food and as a natural medicine in treating hormonal disorders, as an antidepressant, and as an anti-osteoporotic agent. The presented study aims to prove its beneficial cosmetic and chemopreventive properties by testing the antiradical, whitening, cytotoxic, and anticancer properties of differently colored phenotypes that were extracted using three solvents: methanol, water, and chloroform, with the help of the chemometric approach to provide evidence on the impact of single glucosinolanes (seven identified compounds in the HPLC-ESI-QTOF-MS/MS analysis) on the biological activity of the total extracts. The tested extracts exhibited moderate antiradical activity, with the methanolic extract from yellow and grey maca phenotypes scavenging 49.9 ± 8.96% and 48.8% ± 0.44% of DPPH radical solution at a concentration of 1 mg/mL, respectively. Grey maca was the most active tyrosinase inhibitor, with 72.86 ± 3.42% of the enzyme activity calculated for the water extract and 75.66 ± 6.21% for the chloroform extract. The studies in cells showed no cytotoxicity towards the human keratinocyte line HaCaT in all studied extracts and a marked inhibition of cell viability towards the G361 melanoma cell line, which the presence of pent-4-enylglucosinolate, glucotropaeolin, and glucoalyssin in the samples could have caused. Given all biological activity tests combined, the three mentioned compounds were shown to be the most significant positive contributors to the results obtained, and the grey maca water extract was found to be the best source of the former compound among the tested samples.

## 1. Introduction

Herbal cosmetics, with the addition of components of natural origin or entirely based on natural ingredients, are gaining popularity these days. This process is undoubtedly related to increased consumer awareness and the need for safer and natural formulations. Natural products already used in the cosmetic industry are active ingredients of skincare products, haircare products, makeup cosmetics, and perfumes [1,2,3]. The possibility of introducing plant extracts and single compounds that could better act on the skin stimulates researchers to search for novel ingredients that could act beneficially on the skin.

Maca (*Lepidium peruvianum* Chacon syn. *Lepidium meyenii* Walp.) is a Peruvian Andean plant from the Cruciferous botanical family that grows above 4000 m above sea level in difficult weather conditions like low temperatures, intense sunlight, and strong wind [4]. It has been cultivated for the past 2000 years [5]. There are a dozen maca phenotypes that differ with the color of the hypocotyls. The most common are the yellow, red, and black ones. Still, there are also other phenotypes, such as grey and purple, which are characterized by a differentiated composition, and because of this, they show various biological properties [6,7]. Among the Incas, maca was a cure for many ailments, and to this day, it is traditionally consumed by the inhabitants of Peru. Recently, it has been gaining popularity, especially in the dietary supplements market, thanks to its multi-directional action and rich composition in primary metabolites, like vitamins (C, B3, B6, B1, B2, B12, E, A), minerals (calcium, magnesium, sodium, potassium, iron, copper, zinc, selenium), essential amino acids, proteins, carbohydrates, and fatty acids [8], and secondary metabolites—glucosinolanes, alkaloids, flavonoids, anthocyanins, tannins, saponins, and others. Glucosinolanes that have been considered the chemotaxonomic markers and inducers of anticarcinogenic activity of maca are the most represented active ingredients of *Lepidium meyenii* together with the specific macamides and macaenes [5,9,10,11]. Thanks to the presence of N-hydrosulfates, the plant exhibits various biological actions. As previously proven, the administration of maca influences female and male reproductive systems [12,13,14,15] and triggers anti-osteoporotic [16,17], cardioprotective [18], antidepressant [19,20], antiviral [21], antiulcer [5], and antioxidant actions [22]. The latter properties were engaged in the process of tumorigenesis inhibition, which was proven in in vitro tests on leukemia, colorectal adenoma, and liver cancer cells [23].

Several sources denote the plentiful sulfur-containing metabolites in the representatives of the Brassicaceae botanical family. In the case of maca, several structures were identified by HPLC-MS and NMR techniques, but among them, glucotropaeolin was found to be the leading metabolite. However, the plant also contains other derivatives from at least two classes of glucosinolanes, including benzylglucosinolates (e.g., glucotropaeolin, glucolimnanthin, sinalbin) and allylglucosinolates (e.g., pent-4-enylglucosinolate) [24].

Next to therapeutic applications, *L. peruvianum* hypocotyls have also proven to have cosmetic properties that interest the authors of this work. Maca was tested on animal skin models, showing wound-healing activity and protection from UV radiation [25]. According to the authors, both properties were induced by glucosinolanes and polyphenols in the extracts [26,27]. Also, polyphenols from maca were found to be beneficial for anti-aging and correcting the appearance of the skin [28].

As maca has been proven to have cosmetic potential in a few studies, this work aims to deliver further evidence of its impact on the skin and the role of single constituents from the glucosinolanes group on the extract’s total activity. The undertaken research tasks implement a complex approach thanks to which the compositional data recorded in the HPLC-MS-based analyses are set together with the results of biological tests and processed using chemometric methods, which allows for a direct linkage of the concentration of single molecules and the final activity of the total extract. Using this innovative approach, we wanted to explain the role of glucosinolanes in the plant and see if, despite their proven therapeutic properties, these sulfur-containing compounds can also be precious for the skin. This study focuses on determining tyrosinase inhibitory potential, antioxidant properties both in vitro and in cell lines, and anticancer action toward skin carcinomas by extracting different polarities from four differently colored phenotypes of *L. peruvianum* growing in Peru. These particular properties have not yet been elucidated for maca extracts, even if they are related to developing severe cosmetic and health problems. There is much evidence in the scientific literature about a direct link between antiradical properties and anticancer activity. Excessive levels of free radicals appearing in an organism exposed to various stressors may constitute a triggering factor leading to the disruption of natural defense mechanisms and the destabilization of genetic material and physiological processes in the human body’s cells. The administration of free radical scavengers, inducers of natural defense systems (e.g., superoxide dismutase, glutathione), or inducers of trace element chelation—administered internally or externally—will regulate an internal physiological balance and counteract the ongoing aging processes or cancerous changes [29], which are aimed to be proven for maca extracts in this study.

Another goal of this work is to find eventual correlations between the tyrosinase inhibitory potential of the samples and anticancer activity. Interestingly, only a few sources discuss the eventual relationship between these two factors. Previously, Pillaiyar et al. [30] proved the pro-oncogenic action of excessively produced melanin, which could support the need to utilize tyrosinase inhibitors on the skin. Based on their observations, this study aims to determine the eventual role of glucosinolanes in tyrosinase inhibition and skin cancer treatment.

## 2. Results and Discussion

### 2.1. Fingerprinting of L. peruvianum Extracts by HPLC-ESI-Q-TOF-MS/MS

*L. meyenii* is a plant rich in natural compounds, in which many groups of primary metabolites have been identified, including vitamins, carbohydrates, minerals, essential amino acids, proteins, fatty acids, and fiber [8], as well as secondary metabolites: glucosinolates, alkaloids, amino acids, flavonoids, anthocyanins, tannins, sterols, saponins, catechins, and prostaglandins [31]. Among them are glucosinolanes, compounds with both sulfur and nitrogen atoms in their structure that can influence skin cells’ functioning. Moreover, the latest scientific research confirmed their valuable anti-proliferative and anti-cancer properties [32].

**Figure 1:**
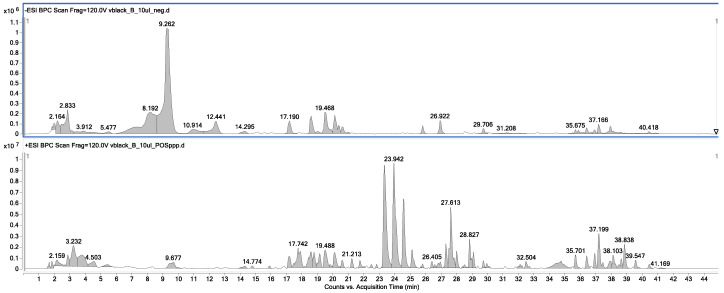
The total ion chromatograms of the black phenotype of *Lepidium peruvianum* were recorded in the negative (**above**) and positive (**below**) ion modes.

The compositional analysis of the obtained extracts allowed us to determine the fingerprint of red, black, grey, and purple phenotypes of *L. meyenii*. The mass chromatograms showing the composition of the extract obtained from the black maca phenotype in the negative and positive ion modes are presented below in Figure 1.

In the positive ion mode, a series of *m*/*z* signals were recorded. Based on the scientific literature, it can be concluded that they represent the metabolites from the groups of macamides, fatty acids, alkaloids, amino acids, flavonoids, anthocyanins, tannins, sterols, saponins, catechins, prostaglandins, proteins, and sugars [8,33]. However, as previously mentioned, *Lepidium peruvianum* is known to be the richest source of glucosinolanes in nature and contains around 1.5–2% of glucosinolanes [6], which makes them the leading components that are responsible for the total activity of maca extracts. As they are known to exhibit antiparasitic, antituberculosis, and anticancer properties [34,35,36,37], these major components of maca extracts were the compounds of interest in this study on cosmetic properties, as they were clearly visible in the chromatograms recorded in the negative ion mode (see Figure 1). Based on these chromatograms, the glucosinolate identity in the four tested extracts was analyzed. Then, their peak areas were collected to be set with the biological properties in the statistical analysis (see Supplementary File, Table S1).

Among the numerous compounds described in the scientific literature, ten were tentatively identified as the ingredients of the studied extracts. Among them, they were glucoalyssin (**7**), glucotropaeolin (**1**), 4-methoxyindolyl-3-hexylhydroxyglucosinolate (**4**), pent-4-enylglucosinolate (**6**), glucolimnanthin (**2**), 4-methoxyglucobrassicin (**3**), and indolyl-5-methylglucosinolate (**5**). The tentative identification of these molecules was carried out based on the high-resolution *m*/*z* measurements, which allowed for the determination of the total glucosinolate formulas with a high probability (the measurement error did not exceed 10 ppm) and on the analysis of their fragmentation spectra (MS/MS), which were compared with the scientific literature and open mass spectrometry databases (Metlin). The tentatively identified molecules are presented in Table 1 below, whereas their structures, together with the fragmentation spectra, are shown in the Supplementary File (Figure S1).

Under the applied fragmentation conditions, glucosinolanes identified in the extract did not show clear fragmentation signals. This demonstrates a high stability of the molecules in the ion source. Huang et al. [33] proved that GTP constituted 80% of the total glucosinolane content and represented the group of aromatic glucosinolanes that were the most abundant subtype of sulfur-containing compounds in maca. Similar conclusions were drawn from this study as well. Glucosinolanes are compounds composed of a sulfated oxime as a moiety, with its C=N group attached to the sulfur atom from the β-thioglucopyranose. Also, the same carbon atom bonds to the structure of a side group that differentiates glucosinolanes and divides them into different subgroups, including aliphatic-bearing fragments often coming from various amino acids or benzenic (typical for *L. peruvianum* that are generated from phenylalanine or tyrosine) or indolic substituents (resembling the tryptophan derivatives). These differently substituted molecules in the electric field turn into negatively charged ions. When losing their substituents under various collision energy settings in the mass spectrometry analysis, they provide characteristic fragments that help in the tentative identification of the molecules from this group [38]. In the spectral analysis of glucosinolanes from maca, the *m*/*z* 259 ion was present in most of the studied cases. This molecular feature may be treated as a marker of these natural products, as it comes from the sulfated glucose group described by different authors before [39]. Interestingly, a highly intensive *m*/*z* of 259 was not typical for all tentatively identified compounds. Similar to the observations of other authors, some of the glucosinolanes delivered other peaks as the significant signals visible in the MS/MS spectra in this study. Among them was an *m*/*z* of 372 from the loss of the methyl sulphoxide moiety, as was the case of **4**. We observed the fragments arising from the detachment of the whole benzenic group for benzenic substituents, as in the case of **2** (*m*/*z* of 259 Da). The detachment of the benzyl ring in GTP (**1**) was also visible. The *m/z* molecular feature of 166 comes from the detachment of 93 Da from the *m*/*z* of 259. It can be seen that the loss of the benzenic moiety came after the detachment of the *m*/*z* of 149. Generally, the detachment of the former moiety—the methyl sulfoxide group—shows more prominent *m*/*z* features, is more typical for these metabolites, and comes first during the fragmentation process.

As proven by other authors, glucosinolanes are the most numerous group of compounds identified in various *L. meyenii* ecotypes regardless of the place of cultivation [40]. The highest quantity determined in the quantitative studies was calculated for GTP, glucosinalbin, glucolimnanthin, hydroxyglucobrassicin, methoxyglucobrassicin, glucoalyssin, glucobrassicin, and glucoubricin, and is consistent with the results of this study. Among other compounds from the same group, p-hydroxybenzylucosinolate,4-methoxyindolyl-3-hexylhydroxyglucosinolate, pent-4-enylglucosinolate, and glucoalyssin are mentioned [41,42,43].

**Figure 2:**
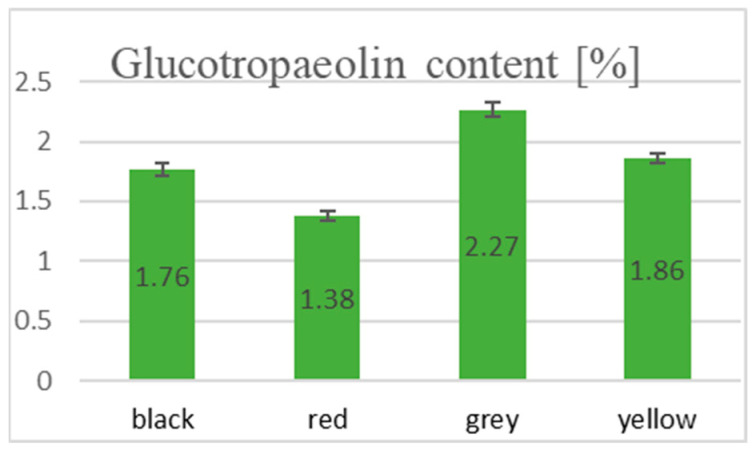
Percentage of glucotropaeolin content in the investigated *L. meyenii* water extracts (n = 3).

This study calculated the percentage of GTP content for all *L*. *meyenii* extracts. Among the tested extracts, water extracts were the most rich in this glucosinolate, with the highest content of 2.26% calculated for the grey phenotype (see Figure 2). GTP was present in the lowest amount of 1.38% in the red phenotype and increased in the black (1.76%), grey (2.27%), and yellow (1.86%) phenotypes.

#### Principal Component Analysis—The Composition of the 12 Extracts

**Figure 3:**
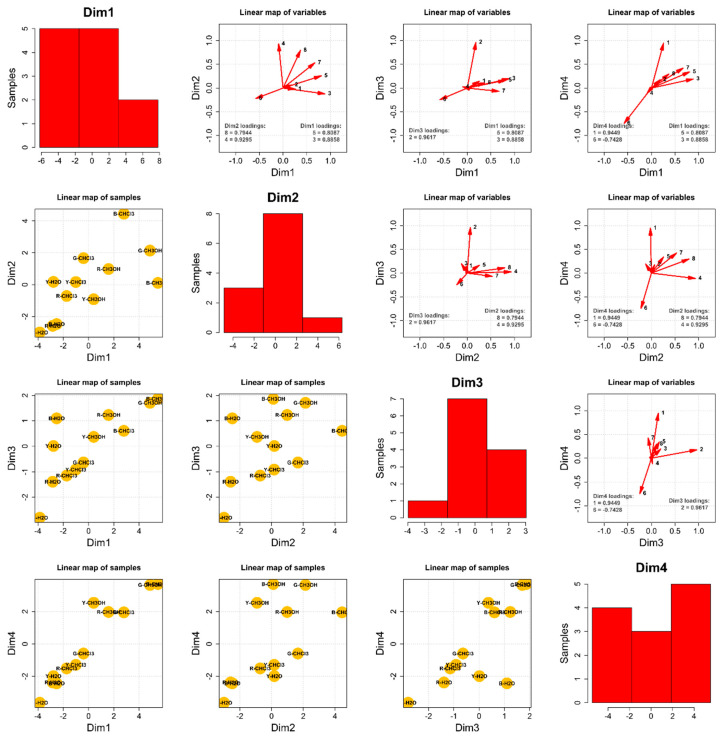
Principal component analysis of the analyzed extracts.

To draw some general conclusions from the performed studies, a thorough chemometric analysis joined the chromatographic analysis results (the peak areas recorded during the experiments). In terms of the composition, the principal component analysis was a four-dimensional problem, as presented in Figure 3. Dimension 1 (Dim1) is defined by indolyl-5-methylglucosinolate (**5**) and 4-methoxyglucobrassicin (**3**), dimension 2 (Dim2) by a derivative of ferulic acid (**8**) and 4-methoxyindolyl-3-hexylhydroxy-glucosinolate (**4**), dimension 3 (Dim3) by glucolimnanthin (**2**), and dimension 4 (Dim4) by glucotropaeolin (**1**) and pent-4-enylglucosinolate (**6**), which were inversely correlated. Information carried by glucoalyssin (**7**) was almost equally distributed between Dim1 and Dim2.

Considering Dim1, it may be stated that methanolic extracts of black and grey species (B-MetOH and G-MetOH), as well as chloroformic extract of the black species (B-CHL), contained the highest amounts of the glucosinolanes 3, 5, and **7**. The chloroformic extracts of red, yellow, and grey species and the methanolic extracts of red and yellow species contained average amounts of the three compounds above. All the water extracts contained negligible amounts of the three discussed substances.

Dim2 analysis showed that the chloroformic extract of black species contained by far the highest amounts of **4** and **8**. The water extracts of red, grey, and black species contained the lowest concentrations of the aforementioned two compounds; the rest exhibited average contents of the discussed metabolites.

Dim3 yielded the most homogenous distribution of the compound glucolimnanthin (**2**) among the 12 studied extracts. Methanolic extracts generally contained higher amounts of **2**. In contrast, water extracts strongly depended on the species, i.e., water extract of black maca (B-H_2_O) contained a high concentration of **2**, whereas water extract of grey maca (G-H_2_O) yielded trace amounts of this metabolite.

Dim4 was perhaps the most radical in the diversification of the studied extracts. It was revealed that all the methanolic extracts and the chloroformic extract of the black species contained the highest amounts of glucotropaeolin (1) and the lowest of pent-4-enyl glucosinolate (**6**). In contrast, the water extract of the grey species (G-H_2_O) contained by far the highest amount of **6**. The rest of the extracts incorporated average concentrations of these two metabolites.

One can notice that the water extract of the grey species (G-H_2_O) is located at the bottom left corner of each of the presented linear maps (Figure 3, lower triangle). This means that, in general, G-H_2_O contained meager amounts of all the studied compounds except **6**. In fact, G-H_2_O incorporated the highest amount of **6** among all the 12 studied extracts. Therefore, water extract of maca grey species might be regarded as a very good source of pent-4-enylglucosinolate. Such a source of **6** is very relevant in the context of the chemometric analysis of biological activity assays of the 12 maca extracts, presented below.

### 2.2. The Bioactivity Determination of the Tested Extracts

#### 2.2.1. Comparative Analysis of the Antioxidant Activity of Maca Extracts

**Figure 4:**
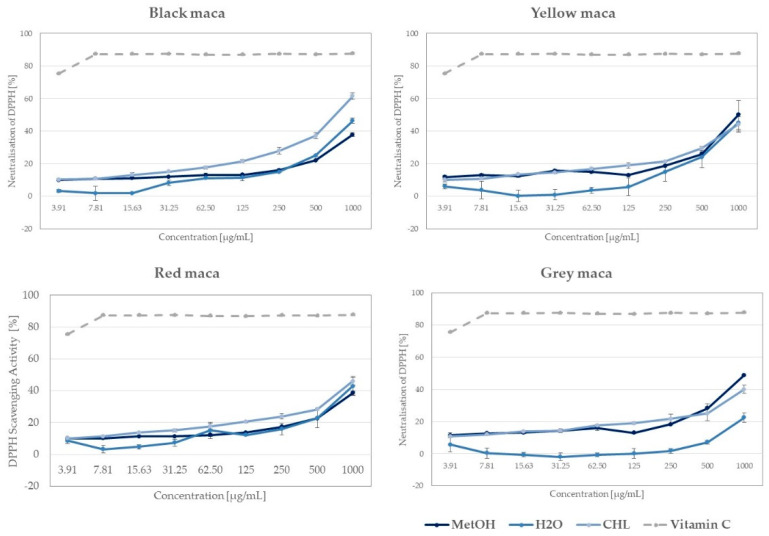
The antiradical potential of differently colored phenotypes of maca and the extracts of different polarity studied in the DPPH in vitro assay (MetOH—methanol, H_2_O—water, CHL—chloroform extracts) in comparison with vitamin C; graphs show mean value ± SD; n = 3.

*L. meyenii* contains antioxidants that scavenge free radicals and protect the cells from oxidative damage. Based on the results of the diphenyl-1-picrylhydrazyl (DPPH) radical scavenging assay, it can be concluded that the extracts from analyzed maca phenotypes were moderately active radical scavengers. The strongest free radical scavenging activity was detected for the extract from the black phenotype of maca extracted using chloroform. The 1000 µg/mL concentration was capable of scavenging 61.36% of radicals (see Figure 4).

The highest concentration of other tested extracts (1000 µg/mL) also showed a marked antiradical potential. Among the most active samples, the methanolic extract from the yellow maca (with 49.9 ± 8.96%), the methanolic extract from the grey phenotype (with 48.8% ± 0.44 activity), the water extract from the black maca (with 46.38± 1.69%), the chloroformic extract from the red maca (with 45.9 ± 2.35%), and the water extract from the yellow maca (with 45.1 ± 5.22%) can be listed. On the other hand, the samples with the weakest antioxidant potential included the grey maca water extract (22.4 ± 2.99%), the methanol extract from the red maca (38.5 ± 1.14%), and methanol extract from the black maca (37.8 ± 1.08%). Interestingly, the solvent did not determine the antiradical potential in this study, and all three types of extracts showed a significant antiradical potential.

Uto-Kondo et al. recently compared DPPH scavenging activity of seven phenotypes of maca grown in Japan, showing that ethanolic extracts from purple maca hypocotyls are more active than the extracts from white, yellow, or red maca [44]. However, these results cannot be directly compared with ours, obtained from plant material collected in Peru, as it is well known that the phytochemical composition of maca and consequently its biological properties strongly depend on the cultivation altitude of this plant [45].

**Figure 5:**
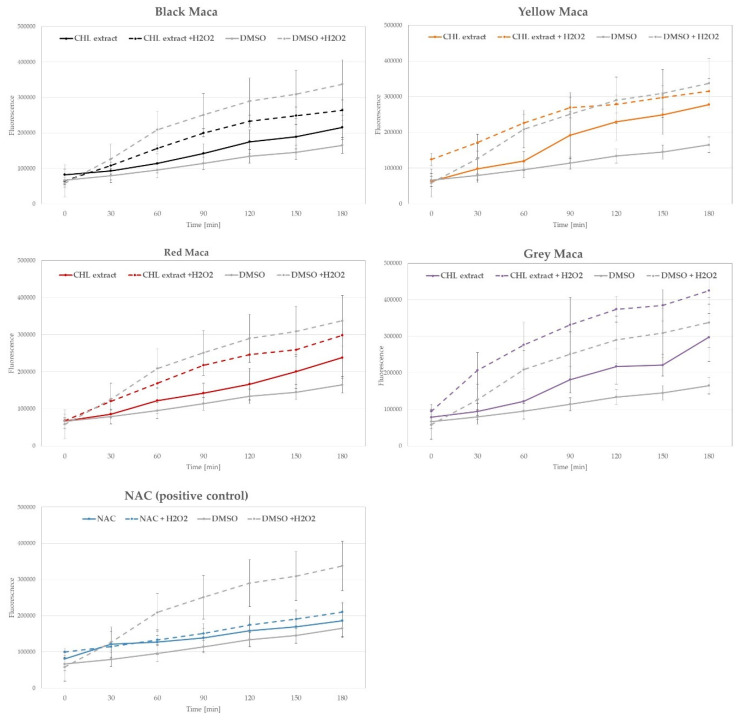
The influence of chlorophormic extract (CHL, 500 µg/mL) from differently colored maca phenotypes on intracellular ROS levels of HaCaT keratinocytes treated with 1 mM H_2_O_2_; 2 mM N-acetycysteine (NAC) was used as a control antioxidant; graphs show representative values (mean ± SD) for one out of three experiments.

Although DPPH scavenging assay is a popular, easy, and affordable approach for the measurement of antioxidant properties of plant extracts and other samples, chemically stable free radicals are not an exact model of radical reactions in biological systems, and therefore, it is important to confirm the antioxidant properties of tested samples also using other experimental approaches. For that reason, the antioxidant activity of extracts from analyzed maca phenotypes was also assessed in vitro, using human keratinocyte cell line HaCaT, loaded with the fluorogenic dye H_2_DCFDA. After the diffusion into the cell, H_2_DCFDA is deacetylated by cellular esterases and subsequently oxidized by ROS into 2′,7′-dichlorofluorescein (DCF). In our study, the generation of DCF in HaCaT cells was monitored over time following the treatment with 1 mM H_2_O_2_ pre-mixed with maca extracts (500 µg/mL). In this analysis, only the CHL extracts were included, as they showed the most significant antioxidant potential in DPPH scavenging assay, and 2 mM N-acetylcysteine (NAC) was used as a control antioxidant. As presented in Figure 5, CHL extracts from black and red maca slightly decreased intracellular ROS levels in HaCaT cells compared with control cells treated with the solvent (DMSO) and H_2_O_2_. However the observed differences were not statistically significant. In regards to yellow maca, the presence of the extracts did not influence the ROS generation following H_2_O_2_ treatment, but the extract alone was able to increase intracellular ROS in HaCaT cells. A similar effect was detected for gray maca. For this sample, the combination of the CHL extract with H_2_O_2_ induced even higher intracellular ROS levels than the treatment with H_2_O_2_ and DMSO. These results further confirmed the differences in antioxidant potential between maca phenotypes of different color and support the significantly stronger antioxidant activity of black and red maca samples. The data obtained from the cell-based studies suggest also that other mechanisms apart from the electron transfer, shown using the DPPH scavenging assay, may be responsible for antioxidant properties of maca phenotypes. For instance, polyphenols and glucosinolanes present in maca extracts were also shown to modulate the activity of antioxidant enzymes such as superoxide dismutase (SOD), catalase (CAT), and glutathione peroxidase (GPx) [46]. Therefore, observed differences in antioxidant activity of extracts from differently colored maca require further investigations.

The confirmation of the ability of maca extracts to scavenge free radicals is particularly important for cosmetic ingredients. Excess radicals have been proven to affect the condition of the skin, leading to the breakdown of collagen fibers to hyaluronic acid, which manifests itself in faster skin aging, the formation of wrinkles, and a decrease in skin turgor, and may lead to the process of carcinogenesis in the long term [47]. Also, an increased antioxidant defense in the skin leads to neutralizing free radicals and the induction of repair processes, which remove the damaged biomolecules. Based on these observations, introducing antiradical ingredients to cosmetics is of the utmost importance to support the native protection mechanisms against free radicals.

#### 2.2.2. Tyrosinase Inhibitory Activity of Maca Extracts

**Figure 6:**
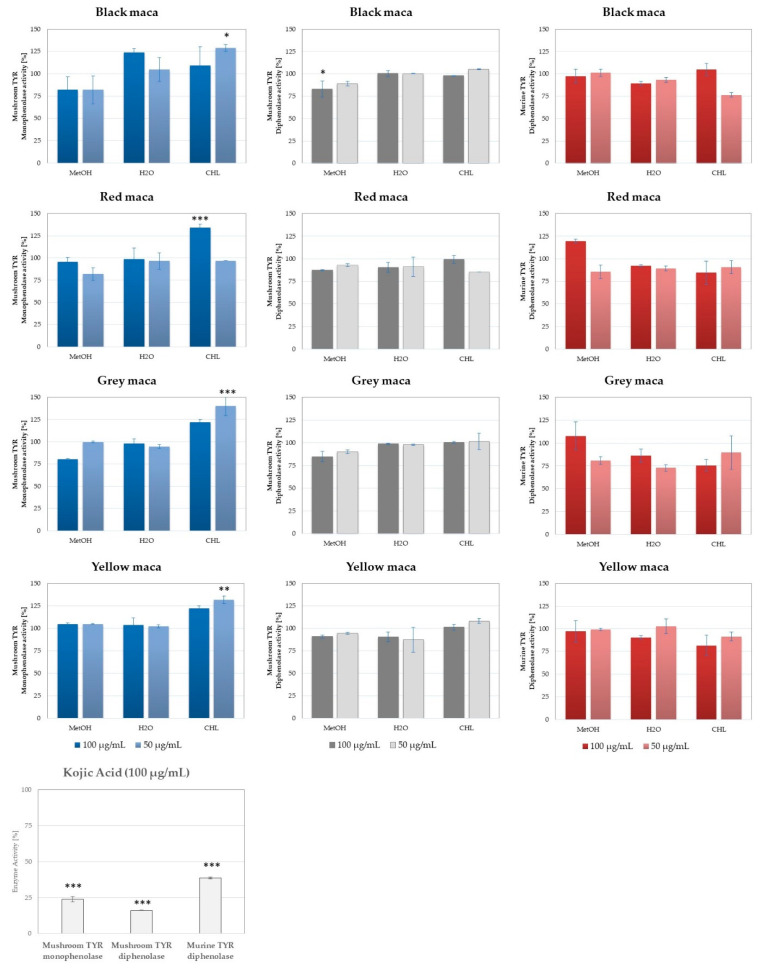
Tyrosinase inhibitory properties of maca extracts studied in mushroom and murine tyrosinase assays—in the left column, monophenolase activity of mushroom tyrosinase; in the middle column, mushroom tyrosinase diphenolase activity; and in the right column, murine tyrosinase and diphenolase activity. Kojic acid was tested as tyrosinase inhibitory control; histograms show mean activity ±SD, n = 3; * *p* < 0.05; ** *p* < 0.01; *** *p* < 0.001.

In the tyrosinase inhibition assay, two concentrations of every extract from the four phenotypes of maca were studied for their ability to interact with the tyrosinase enzyme responsible for melanin formation and skin coloration. For this purpose, two types of enzymes were evaluated—commercially available mushroom tyrosinase and murine enzyme, contained in the lysate of B16F10 murine melanoma cells. In the case of the mushroom enzyme, both monophenolase and diphenolase targeting activities of the extracts were studied using L-tyrosine or L-DOPA as reaction substrates, respectively (see Figure 6). The different approaches shown in this study, which include the evaluation of tyrosinase inhibitory action of enzymes of various kinds, were undertaken to show the eventual differences in the behavior of the studied samples towards the dual activity of tyrosinase enzyme. Mushroom tyrosinase inhibitory assay, even if more often described and more commonly available, delivers results frequently incompatible with mammalian tyrosinase-based determinations due to significant structural and functional differences between murine and mammalian enzymes [30].

As proven by different authors [48], the analysis performed with murine tyrosinase may bring more reliable results. At the tested concentrations, *L. peruvianum* was found to have some whitening potential. At first glance, a similar tendency was observed for both assays targeting the diphenolase-directed activity. However, as presented below in Section 2.2.3, a detailed chemometric analysis of results showed the differences between the results collected in these two tests. The murine tyrosinase was the best target of the constituents from the extracts from grey, black, and red maca. Water extract from grey maca was found to be the most active among all tested extracts (tyrosinase activity reduced to 72.86 ± 3.42%), followed by the chloroform extract from grey maca (75.66 ± 6.21%) and the chloroform extract from black maca (76.5 ± 2.34%). Chloroform was the best solvent to deliver active ingredients for this assay. Yellow and red maca showed the weakest action in the performed tests on murine tyrosinases. However, the activity of the former increased in the mushroom tyrosinase-based test. In the case of mushroom tyrosinase, the obtained results showed a weaker potential. However, water extract from yellow maca, methanol extract from grey and red maca, and methanol extract from black maca were the most potent inhibitors, with a percentage inhibition rate of around 80%. The monophenolase-targeting extracts with the highest activity were the grey maca methanolic extract (80.49 ± 0.61%), methanolic extract from red maca (81.87 ± 7.09%), and methanolic extract from black maca (81.91 ± 15.74%).

To the best of our knowledge, this is the first study comparing the tyrosinase inhibitory activity of extracts from maca phenotypes, but the inhibitory potential of maca towards mushroom tyrosinase was recently demonstrated by Yang et al. [49]. In the mentioned study, mushroom tyrosinase inhibitory potential and melanogenesis inhibition in B16F10 murine melanoma cells was shown for maca extract prepared from commercial root powder dissolved in water (concentration range 2.5–10%). The skin-whitening potential of the extract increased significantly following its fermentation with various *Lactobacillus* strains, surpassing the tyrosinase inhibitory activity of arbutin.

#### 2.2.3. The Analysis of the Tyrosinase Inhibition and Antioxidant Activity Assays by the Chemometric Approach

The heatmap below shows the impact of the individual compounds on the biological activity towards tyrosinase and the antioxidant activity, calculated for the 12 studied extracts, using the linear regression approach. If **X** stands for the matrix of the composition of the studied extracts (i.e., auto-scaled percentage of the individual compounds in a given extract) and **Y** stands for the matrix of the auto-scaled scores in a given biological activity test, the matrix of the coefficients, **b**, can be calculated using formula:

b = (X^T^X)^−1^X^T^Y (1)

Afterwards, each coefficient stored in the **b** matrix was tested for its statistical relevancy, using data stored in the information matrix (X^T^X)^−1^. If the statistical relevancy of a given coefficient was negative, the compound represented by the irrelevant coefficient was removed from the dataset and Equation (1) was solved again, yielding the final result.

In the heatmap below, a positive score for a given compound means that increasing its concentration would increase a score in a given biological activity test; a negative value means the opposite (higher concentration = lower biological activity score). For instance, the coefficient equal to 10.79, displayed by pent-4-enylglucosinolate (**6**) in the mur_diphe_100 test, means that compound **6** greatly contributed to murine tyrosinase + L-DOPA inhibition (while the concentration of the extract was equal to 100 mg/mL). In contrast, the presence of the very same compound significantly lowered the score of all the extracts in the mush_monophe_50 activity test, as displayed by the coefficient equal to −11.13. Zeroes stand for no statistically relevant impact of a concentration of a given compound for the biological activity of the extracts.

**Figure 7:**
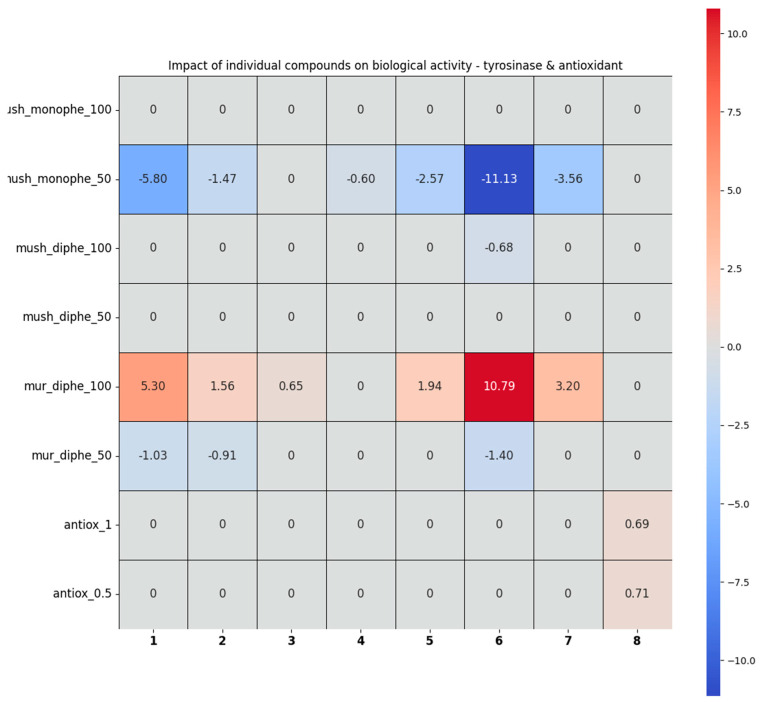
The results of the evaluation of the relationships between the content of individual components and the tyrosinase inhibition and antioxidant properties of the total extract.

As a result of the analysis, several conclusions were drawn on the role of particular glucosinolanes in the biological activity of the total extracts (See Figure 7 and Figure S2). Compounds **1**, **2**, **4**, **5**, **7**, and **6** especially contributed negatively to the mush_monophe test (i.e., they increased enzyme activity), but only in a higher concentration (100 µg/mL); the rest of the compounds in this concentration and all of the ones in the lower concentration (50 µg/mL) were statistically irrelevant. The presence of glucosinolanes **1**, **6**, **2**, **3**, **5**, and **7** (especially the first two) increased the scores of the mur_diphe test (i.e., they decreased enzyme activity), but only in the higher extract concentration (100 µg/mL), whereas none of these compounds made a positive contribution to the test in the lower concentration (50 µg/mL). Also, compounds **4** and **8** were statistically irrelevant in the mur_diphe test, whereas **3**, **5**, and **7** were statistically irrelevant in this test only in the lower extract concentration (50 µg/mL). The magnitude of the negative impact on the mush_monophe test (increasing enzyme activity at 50 µg/mL) was almost the same as the positive impact on the mur_diphe test (decreasing enzyme activity at 100 µg/mL) for compounds **1**, **2**, **5**, **6**, and **7**. Also, in the case of the antioxidant activity, only one compound—**8**—contributed positively, i.e., increased the free radical scavenging in both concentrations; the rest exhibited no statistically relevant impact, which may explain the occurrence of moderate antiradical properties of the investigated extracts in which glucosinolanes were the major components. After a more in-depth analysis of the obtained results, there was a moderately apparent inverse correlation between mur_diphe and mush_diphe, which was highlighted for the concentration of 100 µg/mL only (Figure S2 in the Supplementary File). The correlation coefficient for c = 100 was R = −0.72, and it passed the *t*-test of correlation significance at the 95% level, i.e., this correlation was statistically significant. On the other hand, the coefficient of determination R^2^ was only 0.52, which means that 52% of the variability of the mur_diphe test could be explained by the mush_diphe test in a linear way, the remaining 48% was due to other sources of variability.

The study on the tyrosinase inhibition, despite the determination of the whitening potential of a sample, can give an idea about the potential application in the treatment of cancer. According to Slominski et al. [50], the inhibition of melanogenesis in melanoma cells either by blocking the active site of tyrosinase or by chelating Cu^2+^ ions sensitized melanoma cells towards cytotoxic action of cyclophosphamide and amplified the immunotoxic activities of IL-2-activated lymphocytes. This important finding opens the door for broader applications of tyrosinase inhibitors not only in the disturbed melanin production but also as a support to anticancer therapeutical strategies.

#### 2.2.4. Anticancer Activity of Extracts towards Skin Cancer Lines

**Figure 8:**
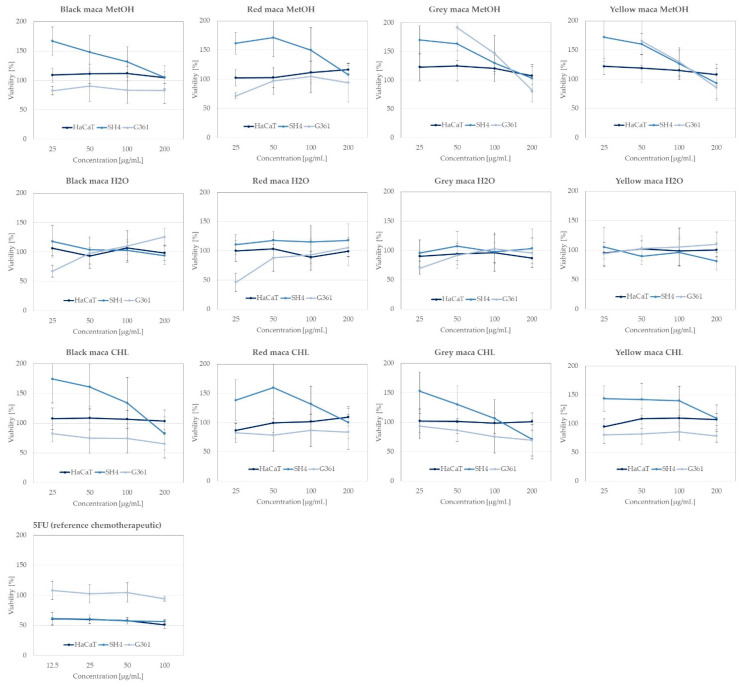
The results of the cell viability assay of the tested *Lepidium meyenii* methanol (MetOH), water (H_2_O), and chloroform (CHL) extracts on the following cell lines: HaCaT keratinocyte, SH4, and G361 melanoma cells following 48 h of treatment; 5′-fluorouracil (5FU) was used as the control chemotherapeutic agent; graphs show the mean viability ±SD; n = 3.

The cytotoxicity tests performed on the *L. meyenii* extracts are important elements determining the safety of the tested extracts and the biological activity. The results of the performed analyses of the selected extracts from different phenotypes of *L. meyenii* are presented in Figure 8 below.

These results are of particular importance especially nowadays, when the number of skin cancer cases is growing, especially in residents of countries with high insolation. The most important etiological factor of this disease is ultraviolet (UV) radiation emitted by the sun [50,51,52] as well as by various other sources, e.g., sunbeds. It is worth noting that skin cancer is the most common malignant neoplasm in the world; however, thankfully, its detection rate is constantly increasing. The most common skin cancers are basal cell carcinoma (BCC) and squamous cell carcinoma (SCC). Other malignancies, such as melanoma, are more rare, but they were selected for this study, as they constitute the group of diseases responsible for the highest number of deaths of all skin cancers [53,54]. Melanoma, as a malignant neoplasm of the skin of non-epithelial origin deriving from neuroectodermal melanocytic cells, has a poor prognosis and is characterized by frequent metastases. In the past decade (2010–2020), the number of invasive melanoma cases diagnosed annually increased by 47% and was the cause of the highest number of deaths [55].

The previously published data show a marked potential of maca extracts and its metabolites in cancer treatment, and that is why the obtained extracts from different phenotypes of maca were tested for their activity against melanoma cells (SH4 and G361 cell lines) to evaluate their potential for treatment of this dangerous type of cancer and towards the immortalized human keratinocyte line HaCaT to check the eventual toxicity of maca metabolites (see Figure 8 and the Table S2 in the Supplementary File).

As presented in Figure 8, none of the extracts decreased the viability of HaCaT cells, which confirms their low toxicity to the normal cells at the tested concentrations and supports their safety.

In relation to the anticancer activity of the tested phenotypes of maca and their different extracts against melanoma, the most promising were the chloroform extracts of differently colored phenotypes. Black maca extracts at the highest tested concentration decreased the viability of the G361 cell line up to 65.32 ± 23.9%, red maca up to 83.91 ± 30.17%, grey maca up to 69.29 ± 26.41%, and yellow maca up to 78.51 ± 10.71%. The G361 cell line was found to be more sensitive to the maca extract treatment in comparison with the SH4 cell line; however, similar tendencies were also observed for the other cell line. There was no statistically significant difference in the cell viability that was observed for water extracts, but in the case of the methanolic extract from the plant, a small inhibition in the viability of cells was observed in the case of the G361 cell line in the case of the grey and yellow phenotypes (see Figure 8 and the Table S2 in the Supplementary File).

The literature shows that the activity of glucosinolates, which are the main group of active compounds in *L. meyenii*, has been studied, e.g., on the breast (MCF-7 and MDA-MB-231), prostate (PC-3), lung (A-549), cervical (HeLa), and colon (HCT116) tumor cell lines in the MTT test, causing a decrease in cell viability by 50% in concentrations from approx. 33 to 80 µg/mL [56] and on bladder cancer (UM-UC-3) and glioblastoma cell lines (LN229), causing a decrease in cell viability by 50% in concentrations from approx. 110 to 190 µg/mL [57]. The extracts from popular members of the Brassicaceae family such as cabbage, cauliflower, radish, and kohlrabi achieved cytotoxicity at the IC 50 level for breast adenoma cancer cell lines (MCF7) and Dalton’s lymphoma (DL) at the following concentrations: cabbage 192.5, 189.7 µg/mL; cauliflower: 378.7, 398.9 µg/mL; kohlrabi: 389.5, 396.9 µg/mL; and radish: 415.4, 423.3 µg/mL in two different cell lines, MCF7 and DL, respectively [58].

In the case of maca, the isolation of the polysaccharide MP21 from *Lepidium meyenii* shed new light on the application of this plant in the treatment of cancers and confirmed its cytotoxic properties on the HEPG 2 liver cell lines caused by activating macrophages via the NF-κB signaling pathway [59]. Also, macamides derived from *Lepidium meyenii* have proven antioxidant and anti-tumor activities that were studied in leukemia HL-60, lung cancer A549, liver cancer SMMC-7721, breast cancer MCF-7, and colon cancer SW480 cell lines [60]. Recently, some trials confirmed a synergism of action of standard chemotherapeutics and maca metabolites in the treatment of cancer. The polysaccharide extracted from *L. peruvianum* tubers, namely, MPW, was combined with doxorubicin and used in breast cancer models in mice (4T1) and demonstrated a stronger effect than doxorubicin alone [61]. The observed action may be also affected by the presence of glucosinolates in this representative of the Brassicaceae botanical family, as these secondary metabolites were proven to be important factors inducing anticancer and cancer-preventing properties in living organisms [62].

The previously published data show a marked potential of maca extracts and their metabolites in cancer treatment, and that is why the obtained extracts from different phenotypes of maca were tested for their activity against melanoma cells (SH4 and G361 cell lines) to evaluate their potential for the treatment of this dangerous type of cancer and towards the immortalized human keratinocyte line HaCaT to check the eventual toxicity of maca metabolites (see Figure 8 and Table S2 in the Supplementary File).

Based on these data, the extracts from maca may be perceived as potential sources of active metabolites, and possibly, after subsequent studies on single metabolites isolated from the extracts, they could be treated as drug candidates against the group of skin cancers.

#### 2.2.5. Chemometric Analysis of the Results Obtained from the Studies in Cells

**Figure 9:**
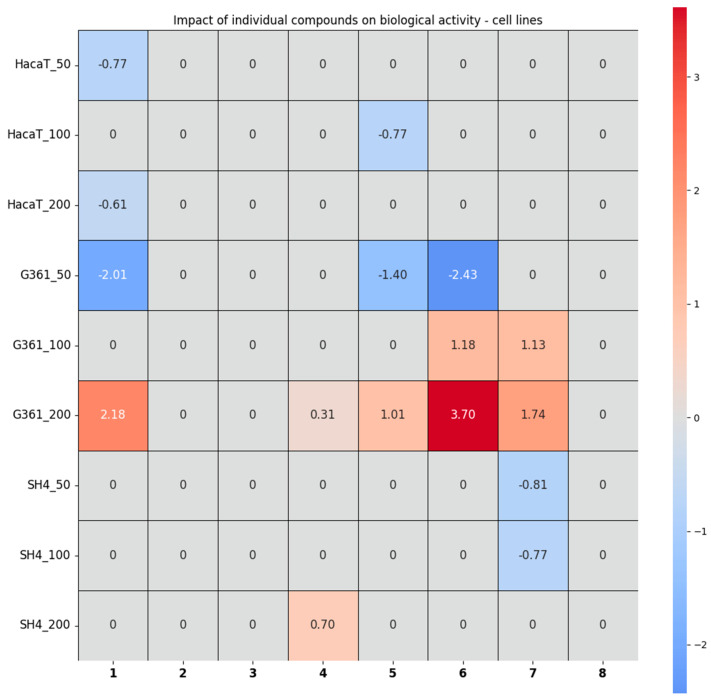
The results of evaluating the relationships between the content of individual components and the impact of the total extract on the tested cell lines—HaCaT, SH4, and G361—at concentrations of 50, 100, and 200 µg/mL. Negative coefficients (blue) state the stimulation of cell growth, and positive coefficients (red) suggest toxicity for cell lines.

In order to better tabulate and compare the cytotoxic and antioxidant properties, the individual impact of compounds **1**–**8** on the tested cell lines was calculated for extract concentrations of 50, 100, and 200 µg/mL, respectively. The heatmap, obtained via regression analysis—as it was presented in the analysis of in vitro assays—is presented in Figure 9 and the data on the constructed model are shown in the Supplementary File (Table S3 and Figures S3 and S4).

The analysis of the results obtained from the assays in the cell lines confirmed a negligibly negative effect of glucotropaeolin (**1**) and indolyl-5-methylglucosinolate (**5**) on the HaCaT cells, which resulted in a slight increase in cell viability. Also, 4-methoxyindolyl-3-hexylhydroxy-glucosinolate (**4**) exhibited a positive impact on the SH4 cell line, which can be translated to a statistically significant reduction in its viability, but only at the highest tested extract concentration of 200 µg/mL. Glucoalyssin (**7**) had a negative impact on the test at the lower concentrations only and increased the cell viability of the SH4 cell line. The remaining compounds exhibited no statistically significant impact on SH4. Concerning the last tested cell line, G361, pent-4-enylglucosinolate (**6**) in particular, as well as **1**, **4**, **5**, and **7**, contributed positively to the G361 test at the highest investigated concentration of 200 µg/mL, i.e., significantly decreasing G361’s cell viability. In the end, pent-4-enylglucosinolate (**6**), glucotropaeolin (**1**), and glucoalyssin (**7**) seemed to exhibit the strongest cell viability, decreasing action towards the G361 cell line.

## 3. Materials and Methods

### 3.1. The Reagents

The reagents used for extraction and chromatographic characterization of the plant, namely, chloroform and methanol, were purchased from Avantor Performance Materials (Gliwice, Poland). HPLC analysis was performed using chromatography-grade acetonitrile, acetic acid (Merck, Darmstadt, Germany), and double-distilled water (Millipore, Temecula, CA, USA). In contrast, the mass spectrometry analysis of the isolate required spectrometric purity water, acetonitrile, and formic acid, which were also delivered by Merck.

### 3.2. The Extraction and Chromatographic Fingerprinting of L. peruvianum Extracts

Black, red, grey, and yellow *L. meyenii* ecotypes were used for the studies. Dried whole tubers from the place of cultivation—the “Pactcha” field, located at an altitude of 4180 m above sea level in the area of Tapu and Tarma, located in the Junin region of Peru—were dried with liquid nitrogen for 40 min and crushed. Three types of extracts were prepared from the tested plant material. Aqueous extracts were obtained by boiling fresh tubers. A total of 100 mL of distilled water were added to 10 g of the dried tubers of various colors and the black variety in the form of a powder and heated for 2 h under boiling, replenishing the water loss. The tubers were subjected to ultrasound-assisted extraction to prepare further extracts of different polarities. Methanol and chloroform extracts were made at 30 °C in three cycles of 15 min each. The extracts were filtered through filter paper, combined, and dried on a rotary vacuum evaporator at 40 °C. As a result of the extraction process, chloroformic, methanolic, and water extracts from black, grey, red, and yellow maca phenotypes were obtained for further analyses.

The HPLC-ESI-QTOF-MS /MS platform from Agilent Technologies (Santa Clara, CA, USA) was used to analyze the composition of *L. meyenii* extracts. An HPLC chromatograph (1200 series) with a Zorbax Eclipse Plus RP-18 chromatography column (150 mm × 2.1 mm; dp = 3.5 µm) was equipped with a degasser (G1322A), binary pump (G1312C), an autosampler (G1329B), a detector with a photodiode array—DAD (G1315D), and a mass spectrometer (G6530B). Agilent MassHunter workstation software (version B.08.00) was used to acquire MS spectra and process the data.

The temperature of the HPLC thermostat was set at 25 °C, and the UV detection wavelengths were 254, 280, 320, and 365 nm. The performance of the UV detector was in the range of 190–600 nm. Chromatography was performed under the following conditions: a 10 µL volume of each sample was injected into the chromatography column, and the chromatographic separation was performed at a flow rate of 0.2 mL/min using a 45 min gradient elution program. Mobile phases comprised eluent A (0.1% formic acid in water, *v*/*v*) and eluent B (acetonitrile solution with 0.1% formic acid added). The mass spectrometer measurements were carried out under the following conditions: gas temperature and shield gas temperature of 350 and 325 °C, gas flow of 12 L/min; capillary voltage of 3500 V and slicer voltage of 120 V; collision energies of 10 and 20 V, skimmer voltage current of 65 V; and nebulizer pressure of 35 psig. The collected spectra were scanned in the *m*/*z* 50–1200 Da range in both negative and positive ionization mode. Two of the most intense signals seen in the TIC spectrum were automatically fragmented to obtain MS/MS spectra. After collecting two spectra for a given *m*/*z* value, the selected signals were excluded for 0.2 min from further fragmentation.

Each tested sample was dissolved in methanol or a 50:50 *v*/*v* mixture of methanol and water before the analysis and adjusted to a concentration of 10 mg/mL. The sample injection was 10 µL each time.

To perform quantitative analyses of glucosinolates in *Lepidium meyenii* extracts, the areas under the peaks of glucotropaeolin, glucosinalbin, 4-methoxyindolyl-3-hexylhydroxyglucosinolate, glucoalyssin, and pent-4-enylglucosinolate were determined for each of the samples. Additionally, a series of dilutions (n = 5) of standards, glucotropaeolin (purity > 95%), and methoxyglucobrassicacin (>95%) (Extrasynthese, Genay, France), were analyzed to determine standard curves.

### 3.3. Cell Lines

Cytotoxic properties of maca extracts were studied on three cell lines: spontaneously immortalized human keratinocyte HaCaT, purchased from Cell Lines Service GmbH, Eppelheim, Germany [63], and human melanoma SH-4 (CRL-7724) and G361 (CRL-1424) cell lines, obtained from the American Type Culture Collection (ATCC, LGC Standards, Łomianki, Poland). The HaCaT and SH-4 cell lines were cultured in Dulbecco’s Modified Eagle Medium (DMEM) supplemented with 10% (*v*/*v*) fetal bovine serum (FBS), and the G361 cells were grown in McCoy’s 5a culture medium supplemented with 10% (*v*/*v*) FBS. All culture media were purchased from Sigma-Aldrich (St. Louis, MO, USA).

### 3.4. Cell Viability Assay

The viability of the HaCaT, SH-4, and G361 cell lines grown in the presence of maca extracts was analyzed using the neutral Red Uptake Test [64]. The cells were seeded onto 96-well plates at 3 × 10^3^ cells density. The overnight culture was followed; the cells were treated with various concentrations of maca extracts (12.5–400 µg/mL) or an equal volume of the solvent (DMSO) as a control for 48 h. Then, the culture medium was removed, and the cells were treated with 33 µg/mL neutral red solution for three hours. The cells were rinsed with DPBS and lysed using an acidified ethanol solution (1% acetic acid, 50% ethanol, 49% H_2_O). The released neutral red dye amount was estimated by measuring absorbance at λ = 540 nm using a FilterMax F5 microplate reader (Molecular Devices, San Jose, CA, USA) at λ = 540 nm. The mean absorbance value for the lysate from control cells was set as 100% cellular viability and used to calculate the percentage of viable cells following the treatment with each maca extract.

### 3.5. Tyrosinase Inhibition by the Tested Extracts

The tyrosinase inhibitory activity of the tested maca extracts was assessed using the enzyme obtained from *Agaricus bisporus* (Sigma-Aldrich) and using the murine tyrosinase that was obtained from the lysate of the murine melanoma cells (B16F10) as previously described by Strzępek-Gomółka et al. [48] and Uchida et al. [60]. In the mushroom tyrosinase assay, the aliquots of 20 μL of the enzyme (500 U/mL) were mixed in the wells with 120 μL phosphate buffer (100 mM, pH = 6.8) and 20 μL of the investigated extracts (50 and 100 µg/mL) and incubated for 10 min at room temperature. Later, 40 μL of a 4 mM L-DOPA (for diphenolase activity) or L-tyrosine (for monophenolase activity) solution was added to the samples and incubated for the following 20 min under the same conditions in darkness. The dopachrome formation was later measured at λ = 450 nm using a FilterMax F5 microplate spectrophotometer (FilterMax F5 Molecular Devices, San Jose, CA, USA). In the murine tyrosinase inhibitory assay, an aliquot of the B16F10 cell lysate with 20 µg protein was used together with 20 µL of the extracts (at concentrations of 50 and 100 µg/mL) and 40 µL of 4 mM L-DOPA (for diphenolase activity), as previously described [65]. At the end, 100 nM phosphate buffer with pH 6.8 were added to fill the mixture up to 200 µL. The samples were later incubated in darkness at 37 °C, and dopachrome formation was measured at λ = 450 nm. The recorded results from both assays were corrected by subtracting a blank that was calculated for the mixtures of the extracts and buffer with no addition of the enzyme. The control samples, corresponding to 100% tyrosinase activity, contained appropriate volumes of all reagents except from the extract. Also, a trial with kojic acid (at 1, 0.5, and 0.25 mg/mL) was performed to obtain a positive control. Each sample was analyzed in 3 independent repetitions. Statistical significance between the control and each experimental sample was analyzed using one-way analysis of variance (ANOVA) followed by Tukey’s test using GraphPad Prism 7.0 Software (GraphPad Software, San Diego, CA, USA).

### 3.6. Antiradical Activity Determination of the Extracts

#### 3.6.1. DPPH Scavenging Assay

The antioxidant activity of maca extracts was assessed using a DPPH radical scavenging assay based on the methodology published by Matejic et al. [66]. For this purpose, 100 of the prepared extracts diluted in DMSO (1–0.002 mg/mL) were mixed with 100 μL of the ethanolic (96% *v*/*v*) solution of DPPH (25 mM) (A540 ≈ 1). The absorbance was measured after 10 min of incubation in darkness and at room temperature at λ = 540 nm using a microplate reader (FilterMax F5 Molecular Devices). Later, the percentage of DPPH scavenging was determined using the following equation:

% of DPPH radical scavenging = [1 − (Abs(S)/Abs(C))] × 100

where Abs(S) is the absorbance of the extract, and Abs(C) is the absorbance of the control sample. Each extract was analyzed in 3 independent repetitions.

#### 3.6.2. Detection of Intracellular ROS Levels—H_2_DCFDA Assay

The intracellular ROS levels in H_2_O_2_-treated HaCaT keratinocytes were measured using a 2′,7′-dichlorofluorescein diacetate (H_2_DCFDA) assay according to the modified protocol by Wu and Yotnda [67]. For each experiment, 10,000 HaCaT keratinocytes were plated per well onto black-walled, 96-well plates and cultured overnight in DMEM medium supplemented with 10% FBS. The next day, the cells were loaded with 5 µM H_2_DCFDA diluted in serum-free, phenol red-free DMEM at 37 °C and 5% CO_2_ for 30 min in darkness. Chloroformic maca extracts (final concentration 500 µg/mL), 2 mM N-acetyl-l-cysteine (NAC), or an equal volume of DMSO (solvent control) were pre-incubated for 60 min in serum-free, phenol red-free DMEM with 1 mM H_2_O_2_ and applied to H_2_DCFDA-loaded cells, and the fluorescence intensity of the formed 2′,7′-dichlorofluorescein (DCF) was measured following 180 min of incubation using a FilterMax F5 microplate reader (Molecular Devices, San Jose, CA, USA) at maximum excitation and emission spectra of 485 and 535 nm, respectively. The obtained values were corrected by the fluorescence of the appropriately diluted maca extracts, NAC or DMSO diluted in serum-free, phenol red-free DMEM (background fluorescence). The experiment was repeated three times, with 5 wells per experimental condition.

### 3.7. Statistical Analysis of Data

All the chemometric analyses and visualizations were performed using R v4.3.0 [68] programming language in RStudio [69] software version 2024.09.0+375 with the pracma [70], factoextra [71], and matlib [72] packages installed. Eight variables (eight compounds) representing the absolute detector responses, i.e., peak areas for a given compound, were auto-scaled to enable principal component analysis (PCA) and regression analysis (RA) (Table S3 in the Supplementary File).

#### 3.7.1. Principal Component Analysis (PCA)

After a standard, formal decomposition of the covariance matrix calculated for the auto-scaled dataset, the first four principal components (PCs) were considered relevant to maximize the information extracted from the original dataset. After selecting the relevant PCs, their vectors were rotated in space to maximize the values of the correlation coefficients between the original variables and the four orthogonal factors using the VARIMAX algorithm, yielding the final four dimensions (Dim) of the presented dataset. The samples’ (maca extracts’) scores in the space of the resulting varivectors (dimensions) were calculated by multiplying the matrix of original variables’ loadings in the space of the varivectors by the matrix of the auto-scaled original dataset.

#### 3.7.2. Regression Analysis (RA)

To estimate the impact of the eight studied compounds contained in maca extracts on the results of biological activity tests of these extracts, regression analysis with the elimination method was applied. For each biological test and the different concentrations of maca extracts used, a model was first established to describe the relationship between the biological activity test results and the composition of individual extracts. In these models, the explanatory variables were auto-scaled detector responses, representing the contents of the eight studied compounds in the extracts. In contrast, the dependent variable resulted from the given biological test.

Next, the quality of the determined model parameters was estimated using the confidence interval method for each model. If the statistical insignificance of a model parameter representing a given chemical compound was detected, the data for the content of that compound were removed from the table of explanatory variables and the entire model was recalculated. Only one chemical compound (the most statistically “insignificant”) was removed in each iteration. The iterations were repeated until only statistically significant parameters remained in the model, i.e., representing compounds with a tangible impact on the biological activity of the given extract in the respective test. The removed parameters, representing compounds that did not affect the biological activity of the extract, were assigned activity coefficients of 0 in Figure 6 and Figure 8.

## 4. Conclusions

Due to their diverse bioactive compounds, natural extracts have garnered significant interest due to their anticancer and cosmetic applications. Many plants contain phytochemicals that exhibit anti-inflammatory, antioxidant, and cytotoxic properties, making them valuable in therapeutic and cosmetic formulations.

*L. peruvianum* emerges as a promising source of bioactive compounds, with strong anti-cancer and health and beauty potential. The phytochemical profile of this plant reveals a rich array of metabolites that exhibit anti-inflammatory, antiradical, or cytotoxic potential, which are crucial to developing therapeutic agents for cancer treatment. Additionally, its beneficial effects on skin conditions suggest that compounds derived from *L. peruvianum* can play a vital role in cosmetic formulations promoting skin regeneration and protection.

This study confirmed the potential cosmetic applications of *L. peruvianum* by assessing its depigmenting effects, antioxidant properties, and anticancer activity against skin carcinomas. The evaluation was performed using extracts of varying polarity derived from distinct phenotypes of *L. peruvianum.* The phenotypes were extracted using three solvents: methanol, water, and chloroform. Given the combined results of all the biological activity tests, the compounds with *m*/*z* values of 371, 408, and 450 were shown to be the most significant positive contributors.

Glucotropaeolin (**1**), the main glucosinolate present in the tested extracts, increased the activity of mushroom tyrosinase + L-tyrosine. It is important to emphasize that most of the eight compounds in the tested maca extracts evaluated by the chemometric approach produced a similar effect at an extract concentration of 50 µg/mL. However, this same compound inhibited murine tyrosinase + L-DOPA at higher concentrations of maca extracts (100 µg/mL). In cell line assays, compound **1** generally stimulated the growth of the HaCaT line and—at low concentrations—also stimulated the G361 line. In contrast, it became one of the main factors inhibiting G361 cell growth at higher concentrations. The primary sources of glucotropeolin (**1**) should be methanolic extracts (from all maca species) and the aqueous extract of black maca.

Depending on the specific test, pent-4-enylglucosinolate (**6**) exhibited the highest positive and negative coefficients. It significantly increased mushroom tyrosinase + L-tyrosine activity at a 50 mg/mL concentration yet strongly inhibited murine tyrosinase + L-DOPA activity at 100 mg/mL. Interestingly, compound **6** generally stimulated the growth of the G361 cell line when maca extracts were added at concentrations of 50 mg/mL. However, the same compound was a major component in reducing cell viability in the two melanoma cell lines when tested at the higher concentration of 200 µg/mL. As presented above, an aqueous extract of grey maca (G-H_2_O) can be considered an excellent source of nearly pure pent-4-enylglucosinolate (*m*/*z* 371). Given its promising but variable biological effects, pent-4-enylglucosinolate (**6**) should be isolated and studied in greater detail.

Glucoalyssin (**7**) overall produced effects very similar to pent-4-enylglucosinolate (**6**), but its impact on tyrosinase inhibition and the cytotoxicity of individual extracts was nearly an order of magnitude lower than that of compound **6**. Glucoalyssin can be isolated from chloroformic and methanolic extracts of different maca species; however, none of these extracts can be clearly distinguished as the best source of **7**.

Regarding methoxybrassicacin (**3**), 4-methoxyindolyl-3-hexylhydroxyglucosinolate (**4**), and a derivative of ferulic acid (**8**), their impact on the tested biological activities was negligible or insignificant, making their further consideration in this context somewhat unwarranted. Glucolimnanthin (**2**) demonstrated notable activity in tyrosinase inhibition assays in a manner nearly identical to compounds **6** and **7**. Yet, its activity was several times lower than that of pent-4-enylglucosinolate (**6**) and noticeably lower than the role of glucoalyssin (**7**). However, compound **2** showed no cytotoxic activity, at least in HaCaT, G361, and SH4 cell line assays.

Considering the results of chemometric analyses in the context of biological activity tests—rather than individual glucosinolates—it should be noted that maca extracts generally increased mushroom tyrosinase + L-tyrosine activity (as clearly shown in Figure 6). However, with regard to murine tyrosinase + L-DOPA, in most cases, the enzyme activity increased slightly at 50 µg/mL but decreased significantly at a concentration of 100 µg/mL of maca extracts. The twelve tested extracts exhibited almost no antioxidant activity at relatively low concentrations. In terms of cell lines, the HaCaT line moderately tolerated maca extracts’ presence (slight stimulation of cell growth). Chemometric analyses suggest that the G361 line showed substantial growth at low extract concentrations (evident stimulation) but suffered dramatically at higher concentrations (high toxicity of maca extracts). Suppose these calculations are compared with the curves presented in Figure 8. In that case, the suggestion appears to be true only for certain maca species, while the data for other varieties suggest the opposite. Furthermore, the values in Figure 9 also indicate that the SH4 cell line was largely indifferent to glucosinolates in the medium. In contrast, Figure 8 shows that this cell line responded quite differently—and radically—to various maca extracts. Hence, it is important to note that the coefficients presented in Figure 7 and Figure 9 represent, to some extent, averages across the twelve extracts, and a value of zero does not indicate a lack of biological activity of the glucosinolate in question—rather, it means that no statistically significant relationship was found between the content of this individual compound and the biological activity assay results, albeit synergistic effects between two or more glucosinolates may not be excluded. Consequently, these coefficients should not be regarded as definitive truths but rather as indications of which glucosinolates may be worthy of further investigations in future studies.

To sum up, the conducted study proved the marked potential of maca extracts to be nature-derived candidates for cosmetic ingredients. However, future research should focus on the isolation and characterization of maca extracts’ bioactive compounds and their detailed mechanisms of action. Clinical studies will be essential to validate the efficacy and safety of *L. peruvianum* extracts in both medical and cosmetic contexts. Integrating natural extracts into anticancer and cosmetic applications offers a holistic approach to health and beauty. To the best of our knowledge, this is also the first study assessing the biological properties of the gray maca phenotype [7].

## Figures and Tables

**Table 1:** The tentatively identified components of the studied extracts from *Lepidium peruvianum* hypocotyls (delta–mass measurement error, DBE—double bonds, and ring number).

No.	Ion (+/−)	Rt (min)	Molecular Formula	*m*/*z* Calculated	*m*/*z* Experimental	Delta (mmu)	DBE	MS/MS Fragments	Proposed Compound
**1**	[M-H]^−^	9.3	C_14_H_19_NO_9_S_2_	408.0471	408.0452	−10.15	6	408.0346; 274.9853; 240.9993; 211.9993; 195.0387; 166.0321	Glucotropaeolin
**2**	[M-H]^−^	12.1	C_15_H_21_NO_10_S_2_	438.0534	438.0550	−3.62	6	274.9855; 259.0156; 243.0192; 195.0305; 145.0588;	Glucolimnanthin
**3**	[M-H]^−^	13.0	C_17_H_22_N_2_O_19_S_2_	477.0643	477.0658	−3.12	8	-	4-Methoxyglucobrassicin
**4**	[M-H]^−^	15.8	C_22_H_36_O_10_N_2_S_5_	551.1733	551.1781	0.16	5.5	389.1287; 370.1942; 341.0926; 317.0991; 309.4013; 283.0377; 261.9778; 235.8406; 216.3231; 193.0498; 134.0416;	4-Methoxyindolyl-3-hexylhydroxy-glucosinolate
**5**	[M-H]^−^	16.5	C_15_H_29_O_10_NS_2_	446.1154	446.1178	−4.0	2	-	Indolil-5-methylglucosinolate
**6**	[M-H]^−^	17.17	C_12_H_24_O_7_N_2_S_3_	371.0946	371.0987	−0.09	11.5	249.0673; 231.0436; 193.0621; 175.0191	Pent-4-enylglucosinolate (Glucobrassicanapin)
**7**	[M-H]^−^	17.6	C_17_H_21_O_10_NS_2_	450.0528	450.0548	−0.04	8.5	437.5571; 417.1699; 386.6801 356.1078; 259.9393	Glucoalyssin
**8**	[M-H]^−^	26.9	C_25_H_20_O_8_	447.1085	447.1082	0.76	16	-	A derivative of ferulic acid
**9**	[M-H]^−^	5.47	C_14_H_19_NO_10_S_2_	424.0378	424.0392	−3.39	6	408.5279; 335.1320; 274.9969; 285.5466; 228.0066; 195.3503	Hydroxybenzyl-glucosinolate
**10**	[M-H]^−^	10.5	C_15_H_21_NO_10_S_2_	438.0534	438.00553	−4.3	6	421.1190; 336.0780; 274.9955; 259.0090;	Methoxybenzylglucosinolate

## Data Availability

The produced data are presented in the manuscript and in the Supplementary Materials.

## Supplementary Materials

The following supporting information can be downloaded at: https://www.mdpi.com/article/10.3390/ijms251910816/s1.

## Abbreviations

mush_monophe	Mushroom tyrosinase + L-tyrosine (monophenolase activity)
mush_diphe	Mushroom tyrosinase + L-DOPA (diphenolase activity)
mur_diphe	Murine tyrosinase + L-DOPA (diphenolase activity)
antiox	Antioxidant activity
HaCaT	Activity towards HaCaT cell lines
G361	Activity towards G361 cell lines
SH4	Activity towards SH4 cell lines
B-MetOH	Black maca methanol extract
B-CHL	Black maca chloroform extract
G-MetOH	Grey maca methanol extract
B-H_2_O	Black maca water extract
G-H_2_O	Grey maca water extract
