# Supplementary material for: Lepidium peruvianum as a Source of Compounds with Anticancer and Cosmetic Applications
Source: Int J Mol Sci. 2024 Oct 8;25(19):10816. doi: 10.3390/ijms251910816 (PMC11476809; doi:10.3390/ijms251910816)
Supplement: Supplementary file 1 [file ijms-25-10816-s001.zip › ijms-3220011-supplementary.pdf]

SUPPLEMENTARY FILE

Lepidium peruvianum as a source of compounds with anticancer and cosmetic applications

Figure S1. The obtained fingerprints of maca extracts of differently colored phenotypes (from the top: black, red, grey, and yellow maca phenotypes) and the MS/MS fragmentation spectra presented for the tentatively identified glucosinolanes from the extracts from *L. peruvianum*

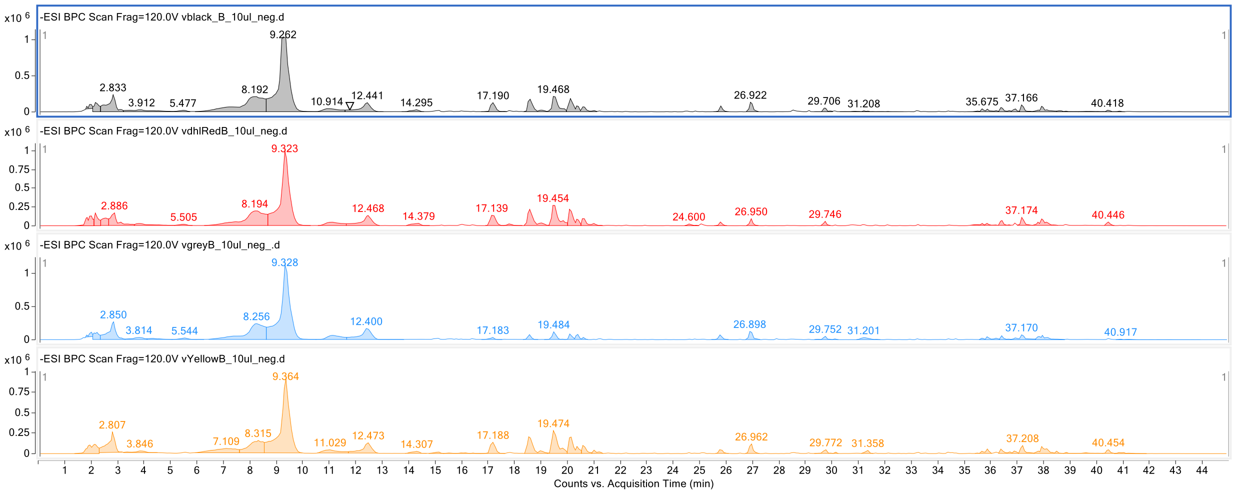

1. Glucotropaeolin

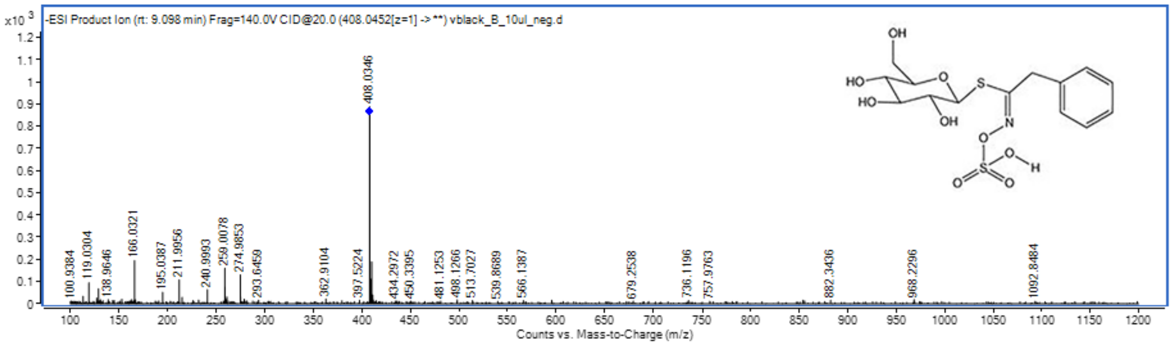

2. Glucolimnanthin

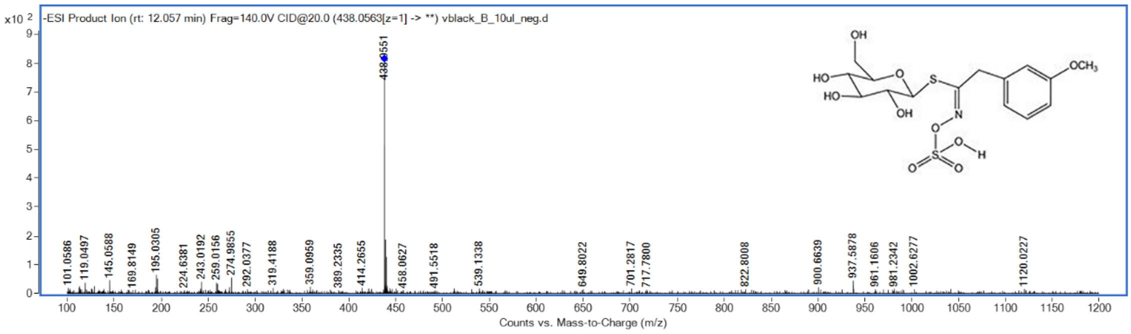

3. 4-Methoxyindolyl-3-hexylhydroxy-glucosinolate

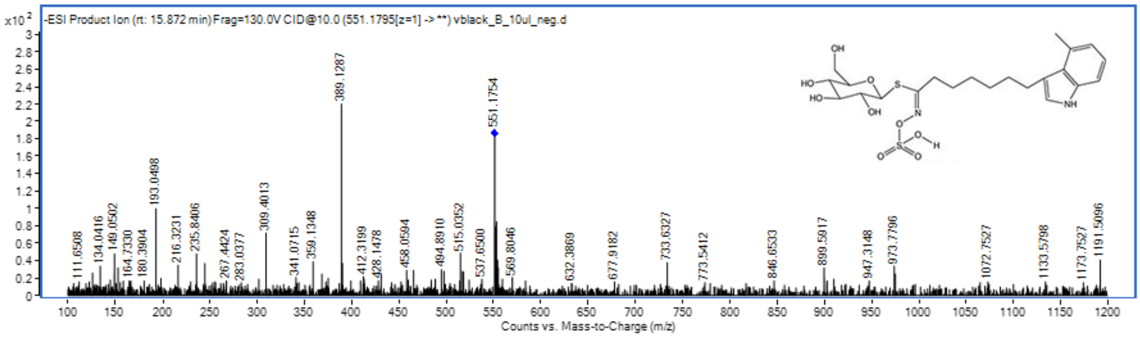

4. Pent-4-enylglucosinolate

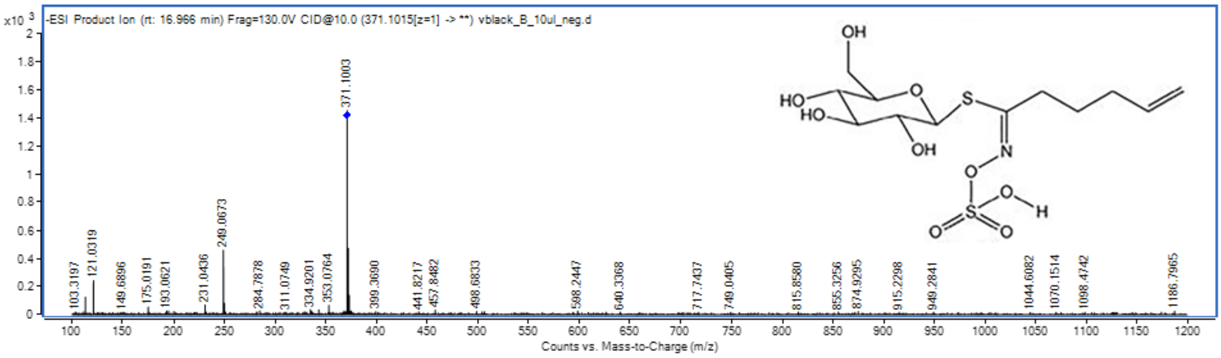

5. Glucoalyssin

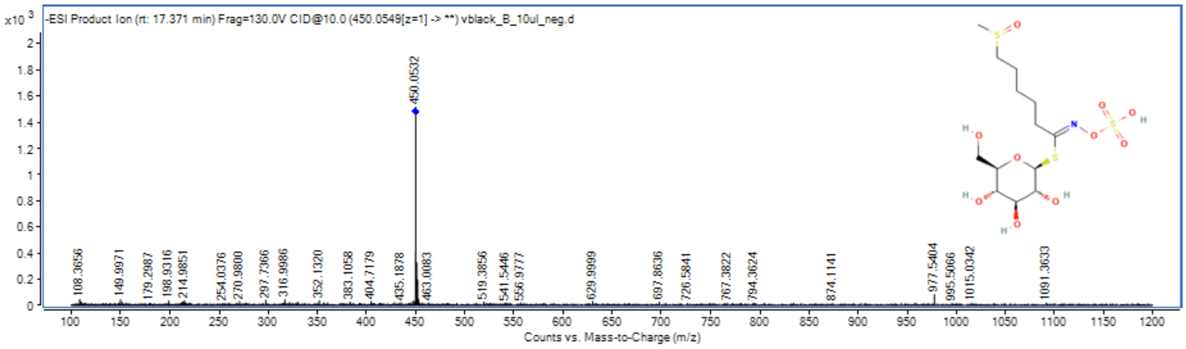

6. Hydroxybenzyl-glucosinolate

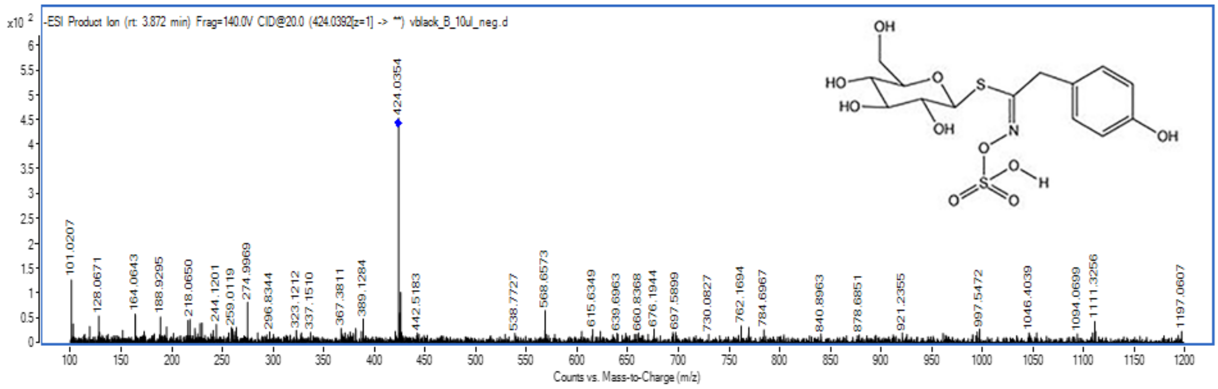

7. Methoxybenzylglucosinolate

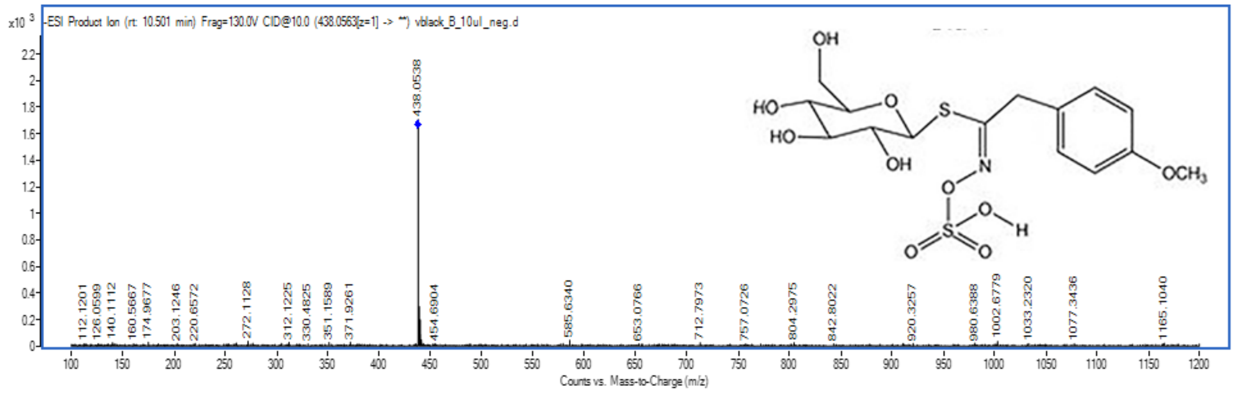

INDIVIDUAL BIOLOGICAL ACTIVITY MODELS – AN EXAMPLE

As a ‘side effect’, each linear regression analysis resulted in a linear dependency model between the concentrations of the individual compounds and a given biological activity test. Each obtained model was statistically relevant and produced viable results, but perhaps the best model was obtained in the case of the cytotoxity test on cell line G361 in regards to extract concentration equal to 200 mg/mL.

Model parameters:

Standard deviation: 0.288
Correlation coefficient (R): 0.979
Determination coefficient (D): 0.958

R and D stand for the coefficient of linear correlation and the coefficient of determination, respectively, determined upon measured biological activity scores and the scores calculated basing on the resulting model.

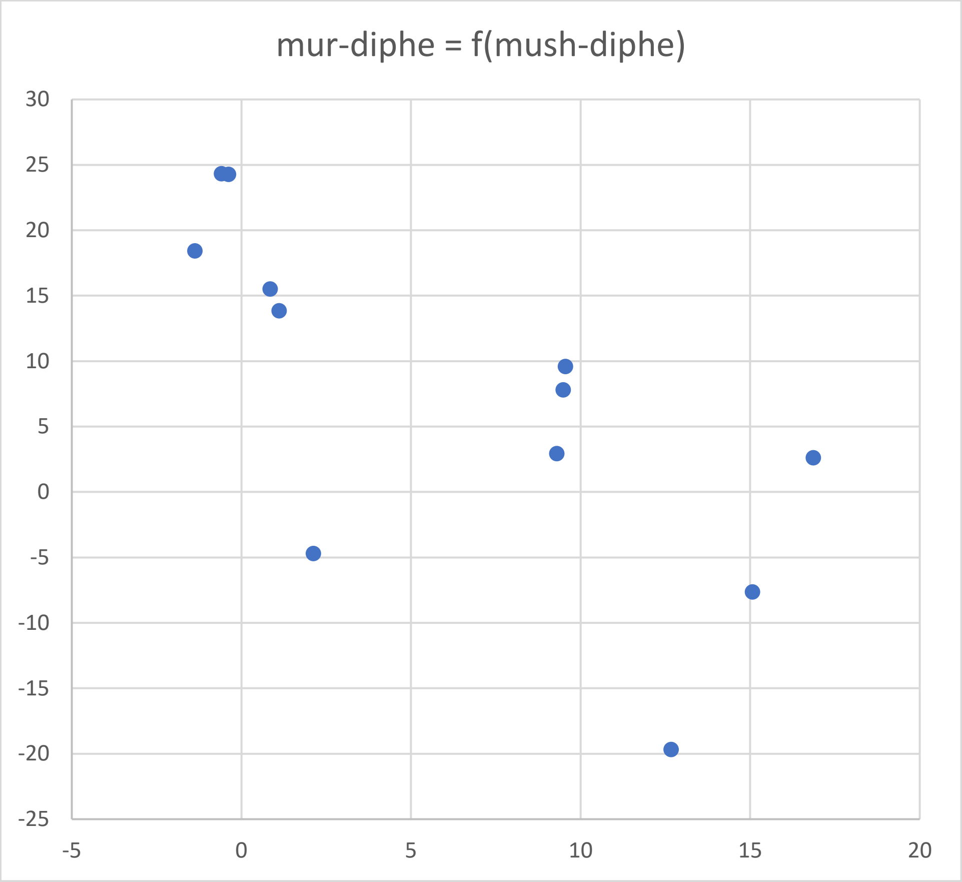

Figure S2. A correlation between mushroom and murine diphenolase activity.

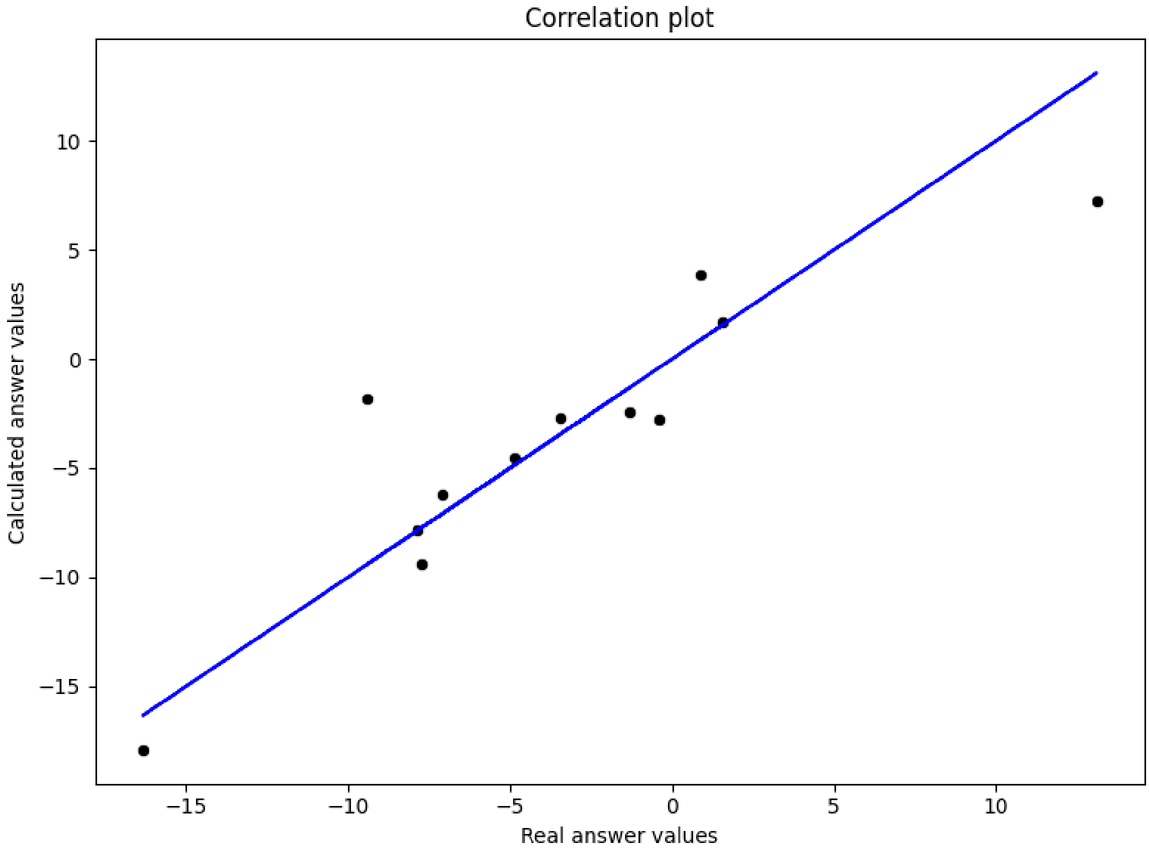

Figure S3. Graphical assessment of the model's quality: correlation plot, displaying the correlation between real biological activity scores and the scores calculated on the basis of the obtained model.

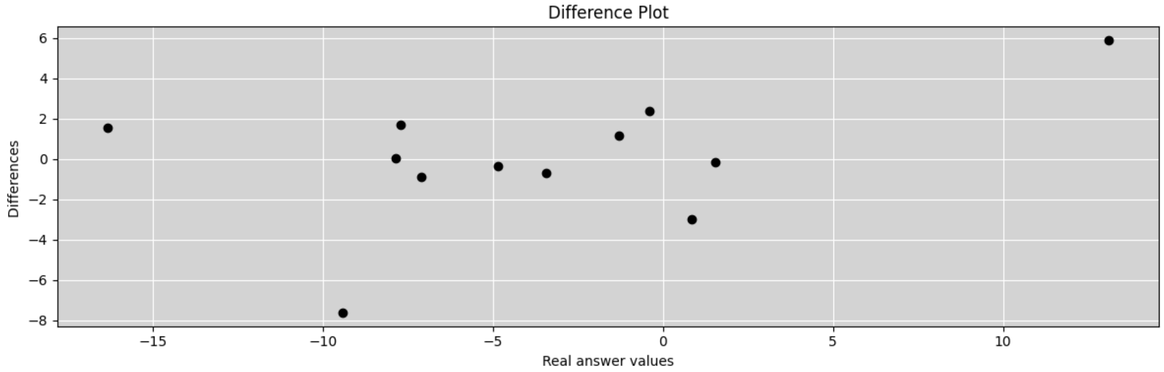

Figure S4. Graphical assessment of the model's quality: difference plot, displaying the differences between measured (real) and calculated biological activity scores as a function of real scores.

Table S1. The raw quantitative data (peak areas) obtained from the chromatographic analyses of the tested extracts, in triplicate.

		**447**	**438**	**408**	**446**	**477**	**551**	**450**	**371**
red chloroform	RCH6-1	1199314	696662	2180295	598891	154638	138907	462032	17276147
	RCH6-12	1425885	752412	2457734	561368	156252	114156	516066	18092741
	RCH6-13	1215770	679012	2287415	601577	150504	128070	490446	17467521
Red water	RH2-1	144835	425032	791373	0	96053	0	242883	10824801
	RH2-12	113071	381558	853047	0	94343	0	201479	9736199
	RH2-13	137870	369314	813760	0	89667	0	250151	9890115
Red methanol	RM1-1	951349	1103469	4452977	848679	519325	145412	1069394	5156612
	RM1-12	935081	985940	4461271	777300	542293	143571	968675	4853115
	RM1-13	858001	1053474		799504	566043	131690	986015	5200171
Grey water	SW2-1	198347	303890	252976	0	0	0	284140	29573987
	SW2-12	221666	322609	238196	0	0	0	345672	31374248
		190152	336055	220527	0	0	0	300672	30005121
Yellow chloroform	YC2-1	1490470	927727	2164112	916930	557542	286282	989091	21026594
	YC2-12	1742180	1073103	2230451	1073992	571692	265630	1107053	22416948
	YC2-13	1552156	986373		950061	560311	226037	1115608	20576415
Yellow water	YH2-1	306022	240614	473779	0	0	61720	16592	3481877
	YH2-12	286468	244888	488586	0	0	56637	0	3808002
	YH2-13	315472	233341	492567	0	0	59501	0	3564512
yellow methanol	YM2-1	1762045	1272293	12008357	579004	447234	0	766159	8857804
	YM2-12	1809682	1327224	12365657	448157	465276	0	1246687	9255635
	YM2-13	1856441	1216890	12647424	515898	420158	0	901531	8706931
Black chloroform	BCL2-1	6132175	1715800	6265804	1809898	2008654	585056	3224709	14126156

	BCL2-12	6047840	1693973	6098174	1738082	1901094	605553	2994273	12835265
	BCL2-13	5831337	1710152	5961712	1819655	2125680	573122	2865519	14501575
black methanol	BM1-1	1978926	2108477	11689607	2331332	1174930	72793	2588486	6625924
	BM1-12	1917043	1957420	9980106	2607462	14479762	75773	2135604	5685223
	BM1-13	1935551	1926466	10402358	2256301				
black water	CW1-1	303535	879910	218005	104782	167933	0	0	11086038
	CW1-2	338485	910723	214603	89505	211959	0	0	9714873
	CW1-13	321178	936080	202156	99604	201752	0	0	9962102
purple methanol	FM2-1	820459	1657441	1802343	3654486	601110	133428	383977	7701563
	FM2-12	886072	1681400	2013378	3928619	696167	156842	411168	7629279
	FM2-13	845456	1610451	1945447	3864022	612287	131015	376198	8006541
purple water	FW2-1	88927	192368	252778	0	0	12119	337753	10562254
	FW2-12	115193	286309	279531	0	0	10952	289514	10661545
	FW2-13	111921	157654	313747	0	0	0	363938	11769089
grey chloroform	GCL-1	2066518	950905	2209304	490800	401313	284320	1442323	16366693
	GCL-12	2311770	908063	2382070	523649	385830	312791	1340593	16421338
	GCL-13	2386440	936353	2420118	515777	432335	296287	1496788	16001575
grey methanol	GM3-1	4819090	1769823	8430172	3274334	2956682	76020	1911923	7011465
	GM3-12	5047211	1952472	8332012	3637765	2853100	83457	2327077	7347297
	GM3-13	5114301	2011351	8450588	3960400	2565417	73698	2222679	7663015
black water - powder	PCH2-1	236307	364658	183941	201301	0	0	422054	9056632
	PCH2-12	228406	359681	250123	196446	0	0	396873	8070553
	PCH2-13	231550	381205	220941	213706	0	0	404085	8624631
purple chloroform	RCH1-1	1818512	1048905	15120133	1672013	679193	150617	980563	18533099

RCH1-12	1875323	1029930	14797127	1760655	666344	139893	1011562	19552532
RCH1-13	1856477	1086146	14130155	1711027	686733	144067	1019751	18101562

Table S2. The results obtained from the cell viability test on different phenotypes of maca presenting the percentage of cells viability upon the treatment with differently coloured phenotypes, at different concentration and using three solvents: methanol, water and chloroform. The dataset was used to sketch the graphs in the 2.4. section of the manuscript.

**Black maca**					
	Concentration	25	50	100	200
HaCaT	**MetOH**	109.155066	111.319013	111.950208	104.85446
		12.2984031	16.3791589	12.4015957	9.89123075
SH4		166.99483	148.011744	131.897569	105.300013
		23.9731626	28.2827671	24.7985281	19.7069294
G361		82.5115056	90.3761719	83.4574106	82.9722762
		7.06962958	25.3780349	22.7859468	22.7471018
	Concentration	25	50	100	200
HaCaT	**H2O**	106.342736	93.4664854	106.947757	98.4615537
		12.8002874	14.5525457	10.2084037	13.1119725
SH4		118.117101	103.784881	103.144066	93.9877176
		26.6165271	22.333874	17.9132462	15.3785644
G361		67.461784	97.8531183	109.779458	125.786931
		10.0147741	25.4824561	27.2229441	14.3552954
	Concentration	25	50	100	200
HaCaT	**CHL**	107.848694	108.846128	106.67594	103.444678
		17.8763839	19.5168494	14.697372	18.9675133
SH4		174.578451	161.208699	134.222225	82.3663049
		40.7337407	37.4378505	43.063866	27.9464111
G361		82.6541755	74.8370026	74.4667215	65.3246452
		14.2294813	25.2528652	24.7253114	23.9690244
**Red maca**					
	Concentration	25	50	100	200
HaCaT	**MetOH**	102.419521	102.72361	111.484531	116.343799

		14.3830809	16.5533047	18.4757204	10.0359222
SH4		161.429183	171.272326	149.853965	108.024082
		19.047019	33.1848056	38.7372508	14.7066255
G361		71.3675214	97.0917353	104.572588	94.2195002
		5.24091609	23.4235192	27.7524394	33.1719612
	Concentration	25	50	100	200
HaCaT	**H2O**	100.013911	103.233416	89.1159151	99.1428096
		17.7438134	16.2951871	10.0159658	23.8986014
SH4		110.681441	118.013913	115.331888	117.800658
		17.4348158	15.1380083	28.2280256	28.1814249
G361		46.1951957	87.9662245	93.3642773	105.351334
		15.5753416	22.8526332	26.477973	14.5187807
	Concentration	25	50	100	200
HaCaT	**CHL**	86.7246196	99.4905458	101.803313	109.419785
		13.0655	16.5897055	13.4747067	14.964184
SH4		138.295566	160.052054	132.138979	100.560228
		34.7392355	60.2567097	30.2549053	26.832419
G361		82.6040581	78.8646998	86.6213856	83.9154502
		16.318165	27.6231983	27.6989995	30.1735498
**Grey maca**					
	Concentration	25	50	100	200
HaCaT	**MetOH**	122.870695	124.978876	120.645483	107.712444
		24.1250886	26.5570342	23.0030225	15.600465
SH4		169.971809	163.398737	130.023821	103.436063
		25.1996035	29.6541207	19.0135627	23.7028202
G361			191.57101	146.732966	82.3613824
			71.503858	31.6152308	20.4971946
	Concentration	25	50	100	200
HaCaT	**H2O**	90.2297385	94.1284754	96.3721983	86.8932159
		7.77512458	18.2730059	16.9857764	8.88963771
SH4		95.5917886	107.212291	97.6813094	103.544465

		22.2982597	24.7439282	32.5908603	32.6991939
G361		69.8369565	91.7196882	102.885242	95.9151376
		10.7013731	22.3704434	24.2605575	25.3747748
	Concentration	25	50	100	200
HaCaT	**CHL**	102.267494	101.447257	98.6665944	101.301269
		20.377593	21.0091162	17.0857449	14.6021977
SH4		153.267978	130.569748	106.7179	71.1888967
		32.3661839	31.4385685	32.4557048	33.3120721
G361		93.4914121	86.5526218	75.4897665	69.2961084
		21.8596348	19.4267843	27.4934603	26.418548
**Yellow maca**					
	Concentration	25	50	100	200
HaCaT	**MetOH**	121.996662	118.966044	115.253982	107.852996
		13.8038801	24.0046976	16.1252673	18.2959938
SH4		172.004037	160.21524	126.533025	93.0701406
		41.2014055	18.1710558	22.336021	25.412118
G361			165.295337	130.076688	85.450975
			41.620226	24.0472458	21.8838914
	Concentration	25	50	100	200
HaCaT	**H2O**	95.2001192	102.469042	98.5684437	100.412608
		9.26034935	13.1144003	24.2378735	12.0292678
SH4		105.148745	89.6976256	96.3214761	81.4776559
		33.1130677	13.9330542	22.8720242	15.0664513
G361		94.0217391	102.983557	105.045543	109.971132
		19.2332799	21.3618866	32.2247257	21.1346428
	Concentration	25	50	100	200
HaCaT	**CHL**	94.5831228	108.642777	109.456413	107.101161
		13.7666208	17.5758007	13.7854683	10.7879882
SH4		143.785125	142.220517	140.004294	109.351048
		22.1807021	28.4066767	25.1443265	23.1284002
G361		80.3590955	81.8245748	85.8141594	78.5087677

		15.2306828	17.9861076	15.1176853	10.710043

Table S3. Model's coefficients

	**coefficient**	**trust radius**	**statistical relevancy**
**408**	2,183868	0,887676	3,573004
**446**	1,011743	0,582539	1,802839
**551**	0,309604	0,269589	0,363192
**450**	1,738338	0,516271	5,792089
**371**	3,698572	1,417053	3,939638
